# Efficient Enantiodifferentiation of Carboxylic Acids Using BINOL-Based Amino Alcohol as a Chiral NMR Solvating Agent

**DOI:** 10.3389/fchem.2020.00336

**Published:** 2020-05-04

**Authors:** Gaowei Li, Minshan Ma, Guifang Wang, Xiaojuan Wang, Xinxiang Lei

**Affiliations:** ^1^School of Pharmaceutical Sciences, South Central University for Nationalities, Wuhan, China; ^2^College of Chemistry and Chemical Engineering, Shangqiu Normal University, Shangqiu, China

**Keywords:** BINOL-amino alcohol, chiral-solvating agents, ^1^H NMR analysis, chiral discrimination, carboxylic acids

## Abstract

A new optically active BINOL-amino alcohol has been designed and synthesized in a good yield and applied as chiral nuclear magnetic resonance (NMR) solvating agent for enantioselective recognition. Analysis by ^1^H NMR spectroscopy demonstrated that it has excellent enantiodifferentiation properties toward carboxylic acids and non-steroidal anti-inflammatory drugs (14 examples). The non-equivalent chemical shifts (up to 0.641 ppm) of various mandelic acids were evaluated by the reliable peak of well-resolved ^1^H NMR signals. In addition, enantiomeric excesses of the ortho-chloro-mandelic acid with different enantiomeric ratio were calculated based on integration of proton well-separated splitting signals.

**Graphical Abstract:**
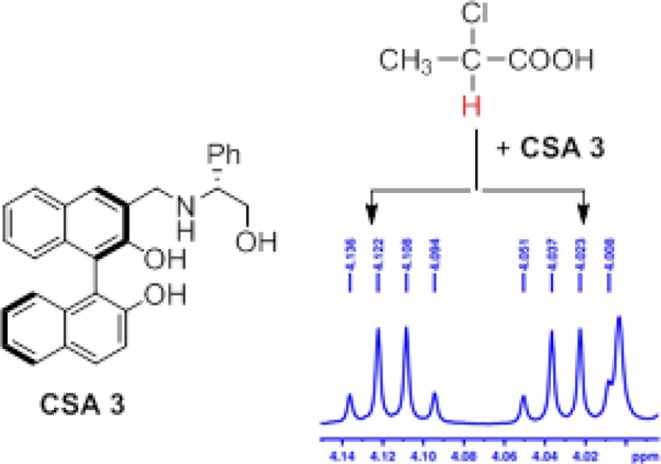
The new optically active BINOL-amino alcohol was used as chiral solvating agent (CSA) for the rapid chiral analysis of carboxylic acids and nonsteroidal anti-inflammatory drugs by 1H NMR spectroscopy.

## Introduction

Chirality plays an important role in chemical, physical, pharmaceutical, and many biological events. The rapid and facial methods to detect and discriminate chiral compounds are highly desirable and urgent to accelerate advance in modern asymmetric synthesis and chiral drug screening (Izake, 2007; Wenzel and Chisholm, 2011a). In this context, the exponentially growing detection demand in this intensive area of research drives the development of chiral analysis (Pu, 2004, 2012; Liu et al., 2010; Nieto et al., 2010; Leung et al., 2012; Cheng et al., 2013; Wolf and Bentley, 2013; Jo et al., 2014; Akdeniz et al., 2016; Yu and Yao, 2017). Among these direct and non-invasive spectroscopic methods of analysis, nuclear magnetic resonance (NMR) spectroscopy plays a leading role and enjoys a special status because it is a reliable, routine technique for monitoring the optical purity and analyzing the absolute configuration of chiral molecules, offering several advantages such as cost-effectiveness, operative convenience, small sample size, and also sensor responsiveness (Parker, 1991; Zalesskiy et al., 2014; Pérez-Trujillo et al., 2015; Silva, 2017; Xu et al., 2019). The general methods to NMR spectroscopic discrimination of enantiomers through chemical shift measurement and spectral splitting observed have been developed: first is to utilize an enantiomerically pure chiral derivatization agent taking advantage of a reactive moiety of the substrate to produce two diastereomers. However, the chiral derivatization agents require cumbersome and time-consuming synthetic procedures and may cause concerns of kinetic resolution and racemization (Seco and Riguera, 2015). The second, chiral-solvating agents (i.e., CSAs) or chiral lanthanide shift reagents (i.e., CLSRs) can form two NMR-observable diastereomeric complexes/mixtures with guests via non-covalent interaction (Wenzel and Wilcox, 2003; Seco et al., 2004; Pérez-Trujillo et al., 2013). In recent years, chiral liquid crystals are also employed for spectral enantiotopic discrimination due to the effect of magnetically induced anisotropic interactions (Lesot et al., 2015; Farjon and Giraud, 2018). The CLSRs in the analyte solution are similar to CSAs with regard to the non-covalent interactions, but the major problem encountered in the use of CLSRs is enormous line broadening due to the paramagnetic interaction in poor resolution and sensitivity (Yang et al., 2005; Wenzel, 2012). In this regard, using CSAs as NMR-observable sensors for structural recognition has significant advantages over others mentioned above. Importantly, only a little amount of hosts (CSAs) and guest are needed without tedious derivatization and purification steps in test samples (Wenzel, 2007; Wenzel and Chisholm, 2011b; Chaudhari and Suryaprakash, 2012; Seco et al., 2012; Uccello-Barretta and Balzano, 2013a,b). In addition, it is not necessary to construct a calibration curve by using enantiopure samples compared with other spectroscopic apparatus, such as fluorescence spectroscopy and circular dichroism. Furthermore, the analytes are readily recovered because of non-covalent interactions, which is very important for difficult-access pharmaceutical compounds or drug samples (Holzgrabe et al., 1999; Uccello-Barretta and Balzano, 2013a,b). Thus, the further development of effective CSAs is highly desirable.

In recent years, various representative types of CSAs have been reported, such as Zwitterionic phosphorus heterocycles (Sheshenev et al., 2013), tetraaza macrocycles (Feng L. et al., 2018; Feng S. et al., 2018), C_2_-symmetrical bisthioureas (Chen et al., 2018), chiral squaramides (Yang et al., 2018), Kagan's amides (Jain et al., 2018), and so on. The binaphthyl-type and related compounds have been widely investigated in asymmetric catalysis, enantioselective fluorescence recognition, and new materials. The chiral binaphthyl units and multiple hydrogen bonding sites containing hydroxyl, or amino groups, can provide an excellent candidate for chiral receptor sensors development (Yu and Pu, 2015; Pu, 2017), especially, they are broadly applicable CSA. For instance, commercially available (R)- or (S)-BINOL and derivatives as chiral-solvating agents to assign the enantiomeric excess (ee) of enantiomeric hydroxy carboxylic acids, synthetic drugs, natural alkaloids, or flavanones via ^1^H NMR spectroscopy (Ardej-Jakubisiak and Kawecki, 2008; Freire et al., 2008; Klika et al., 2010; Redondo et al., 2010, 2013; Chaudhari and Suryaprakash, 2013; Mishra et al., 2014; Yuste et al., 2014; Borowiecki, 2015; Du et al., 2015; Yi et al., 2016; Monteagudo et al., 2017) and bifunctional BINOL-macrocycles containing diacylaminopyridine moieties were developed by Ema et al. (2007, 2008, 2018); BINOL-derived disulfonimide extends the concept of CSA sensing to chiral recognition of O-heterocycles (Couffin et al., 2014); the crownophane and strapped calix[4]pyrrole containing built-in chiral BINOL were used for the enantioselective recognition of chiral amines and carboxylate anions, respectively (Tokuhisa et al., 2001; Miyaji et al., 2007). Chiral BINOL Brönsted acids were selected for determination of various indoloquinazoline alkaloid-type tertiary alcohols and various 3-arylquinazolinones (Liu et al., 2017; Wu et al., 2018), binaphthalene skeleton ureas as sensor for scanned various sulfoxides, phenylethanol, and arylpropanoic acids (Holakovský et al., 2015; Curínová et al., 2018, 2019). The results above indicated that highly active binaphthyl scaffold receptors containing multiple binding units could be used as an extremely versatile reagent for various analytes, and the large atropisomeric naphthyl rings also caused shielding effects through π-stacking stabilization that account for enantiomeric discrimination.

The designed, synthetic new hosts that are capable of discriminately more substrate are often challenging and an important goal for prochiral substrates and have attracted increasing attention in recent years. However, most of existing CSAs are not usually practical because of the splitting of chemical shift non-equivalences too weak to realize baseline resolution, thereby hampering the chemical analysis. Therefore, the development of new CSAs for NMR chiral analysis is still highly desirable. In the last years, our group has successfully developed a different class of CSAs for the determination of enantiomeric ratio and the application of enantiodiscrimination (Lei et al., 2010; Liu et al., 2011; Bai et al., 2019). Among reported CSAs, chiral amino alcohols are especially suitable to be used as chiral sensors as they pose proper nature of non-covalent interactions with substrates. In our previous study, we found that pyrrolidine-functionalized BINOL could be used as a highly effective chiral sensor for the resolution of discrimination and measurement of carboxylic acids. Recently, the simple β-amino alcohol was also developed as a CSA for discrimination of the signals of some carboxylic acid molecules (Ma et al., 2012; Li et al., 2016). In order to know the incorporation of BINOL-derived scaffold in the CSA enantiodifferentiation capacity, we decided to design and explore the possibility to introduce monosubstituted amino alcohol by choosing attached hydroxyl and amino groups with an aim to form multiple hydrogen bonding in the form of π-stacking. Based on this goal, the enlargement of the enantiodistinctive capacity of target BINOL-derived amino alcohol depended on incorporated structural modification that was generalized and developed. In addition, taking it into account commercially available and relatively cheap chiral amino alcohols, we decided to synthesize our target CSA of BINOL derivatives with chiral phenylglycinol as an attached side chain. The convenient and powerful CSA containing chiral phenylglycinol can be utilized to carry out the enantiodifferentiation of carboxylic acids based on well-resolved splitting signals by ^1^H NMR spectroscopy. Herein these results are reported.

## Results and Discussion

**Scheme 1:**
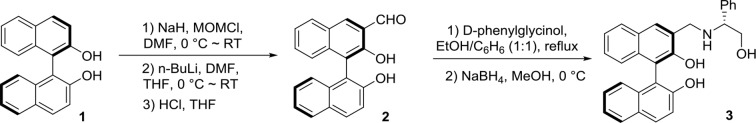
Preparation and structures of 3-monosubstituted BINOL-amino alcohol **3**.

The chiral monosubstituted BINOL-amino alcohol can be readily carried out in five-step sequence according to the reported procedures starting from commercially available (R)-BINOL **1** (Matsunaga et al., 2000; DiMauro and Kozlowski, 2001; Dong et al., 2012; Xu et al., 2016). The key BINOL monoaldehyde was readily generated by lithiation, acylation of the bisprotected BINOL, and cleavage of the MOM ethers starting from the source of commercial (R)-BINOL; subsequently, the requisite monoaldehyde **2** was condensed with ready d-phenylglycinol and followed by reduction with NaBH_4_. The 3-monosubstituted BINOL-amino alcohol **3** was obtained as a yellow solid in 89% yield. The synthetic route leading to chiral 3-monosubstituted BINOL-amino alcohol **3** is shown in Scheme 1 (the general synthesis procedure is illustrated in Scheme S1 and details of all NMR spectras are provided in Figures S1–S10).

**Figure 1:**
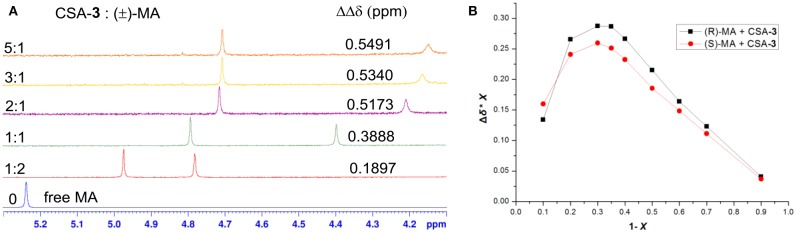
**(A)** Overlaid partial ^1^H NMR spectra and nonequivalent chemical shifts of α-H of (±)–mandelic acid (MA) with various molar ratio in the presence of CSA **3** in CDCl_3_ at room temperature. **(B)** Job plots of CSA-**3** with (R)-MA and (S)-MA. Δδ stands for the chemical shift change of the α-H proton of (R)- and (S)-MA in the presence of CSA-**3**. X stands for the molar fraction of the CSA-**3** (*X* = [CSA-**3**]/[CSA-**3**] + [MA]). The total concentration is 10 mM in CDCl_3_.

With the desired synthetic host in hand, to investigate the discriminating ability of BINOL-amino alcohol **3** as a CSA for the analysis of carboxylic acids, we first performed ^1^H NMR experiment of the racemic mandelic acid (MA) as a test sample in 0.5 mL CDCl_3_. The results of these experiments are shown in Figure 1; the addition of CSA **3** to racemic MA in CDCl_3_ caused non-equivalence C^α^H proton resonance of MA to shift up-field in the ^1^H NMR spectrum; the good signal resolution was collected. The observed two peaks suggest that the host compound was able to interact with racemate guests to convert the enantiomer into different diastereomeric complexes. To find out the suitable stoichiometries of the host–guest complex, the regarding chemical shift ΔΔδ value of C^α^H resonance ranged from 0.1879 to 0.5491 ppm (93.95–274.55 Hz), when the molar ratio of CSA **3** and racemic MA varied from 1:2 to 5:1. By considering cost-efficiency, we know commercially available hosts are often very expensive, and the discriminating ability of **3** to resolve enantiomers at the host:guest molar ratio 2:1 is a clear improvement as a minimum of 1.0 eq. (and in some cases an excess up to 24 eq.) of the host is needed to obtain a maximal resolution (Ema et al., 2007; Uccello-Barretta and Balzano, 2013). From the above detailed analysis, therefore, the molar ratio of 2:1 was finally utilized to select the application in the NMR differentiation of MA derivatives. The stoichiometry of host–guest complex was also determined according to Job's method of continuous variation. As shown in Figure 1B, it showed a probable maximum at 1 – *X* = 0.35; this indicates that CSA 3 and the acid bind in a 2:1 complex under these conditions.

With optimized conditions in hand and encouraged by above satisfactory enantiodiscriminating results, next, we examined the scope of other derivatives of MA. The structure, ΔΔδ values of examined guests, and related spectra are displayed in Table 1. Because of the multiple hydrogen–bond interactions of OH/NH moiety and incorporated anisotropic aromatic group, the CSA associated with all tested aromatic carboxylic acids through ion-paring interaction and exhibited good baseline resolution for large-enough α-H signals on a 500-MHz instrument (Table 1, entries 1–7 and Figures S11–S17). As a whole, these carboxylic acids with a para- or meta-substituent on the phenyl group gave higher ΔΔδ values than those bearing ortho-substituted ones, the ^1^H chemical shift non-equivalences of methane protons reached hundreds of Hertz. In light of the above observation, the para-substituted aromatic carboxylic acids (Table 1, entries 2–4) almost showed good baseline resolution and much bigger ΔΔδ value compared with the ortho-substituted aromatic carboxylic acids (Table 1, entries 6–7); in particular, the MAs with strong electron-donating groups (F-, CF_3_-) gave better results (0.582 ppm, 291.0 Hz and 0.641 ppm, 320.5 Hz; Table 1, entries 2 and 4). However, the ortho-substituted group on the MAs displayed weaker values (0.582 ppm, 291.0 Hz vs. 0.389 ppm, 194.5 Hz, Table 1, entries 2 and 6); ortho-chloro-MA displayed similar enantiodiscriminating ability (0.329 ppm, 164.5 Hz; Table 1, entry 7); the above results indicated that the discriminating ability of CAS **3** could be weakened presumably due to being more sterically hindered in ortho-substituted MAs. However, meta-difluoro–substituted aromatic carboxylic acid showed the relatively bigger ΔΔδ value as 0.592 ppm compared with the ortho-fluoro–substituted one (Table 1, entry 5). The results suggested the stronger electron-withdrawing effects, the larger the corresponding ΔΔδ values. In order to further explore enantiodiscriminating abilities of CAS **3**, the α-methyl protons of the carboxylic acids were also discriminated by the corresponding host only moderately (0.047 ppm, 23.5 Hz; 0.050 ppm, 25.0 Hz; 0.041 ppm, 20.5 Hz; and 0.033 ppm, 16.5 Hz; Table 1, entries 8–11 and Figures S18–S21). We can observe a minor separation of the CH_3_ proton signal when using propionic acid derivatives instead of phenylacetic acid derivatives (Table 1, entries 9 and 11).

**Table 1:** Non-equivalence chemical shift (ΔΔδ) and partial spectra of racemic carboxylic acids (guests) in presence of receptor by ^1^H NMR (500 MHz) in CDCl_3_ at 25°C.

**Entry**	**Guest^a^**	**ΔΔδ (ppm)^b^**	**ΔΔδ (Hz)**	**Spectra**
1	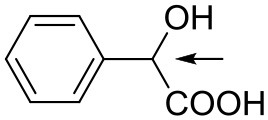	0.517	258.5	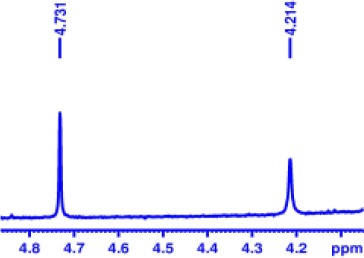
2	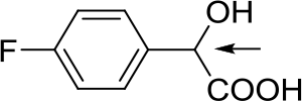	0.582	291.0	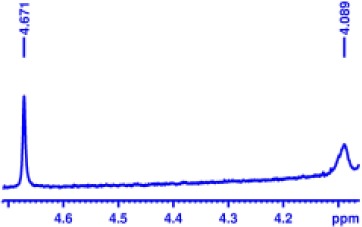
3	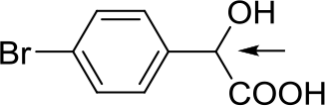	0.522	261.0	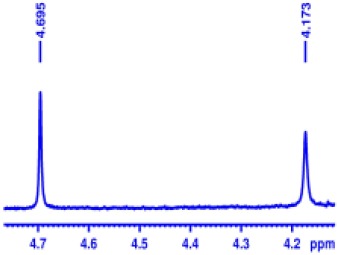
4	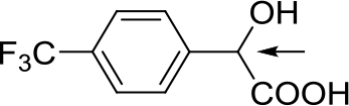	0.641	320.5	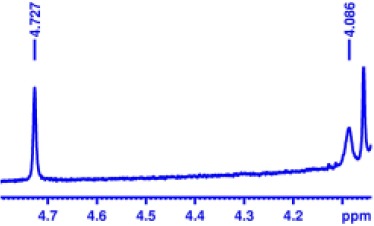
5	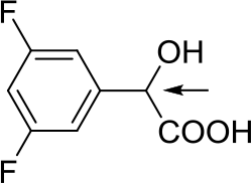	0.592	296.0	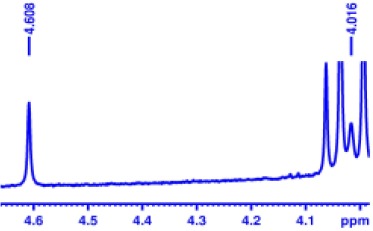
6	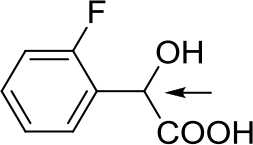	0.389	194.5	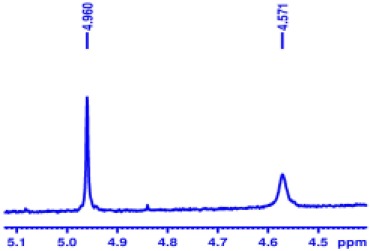
7	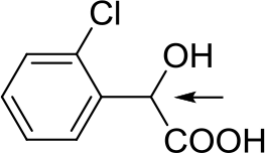	0.329	164.5	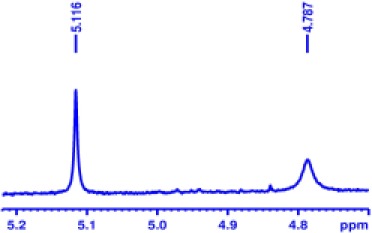
8	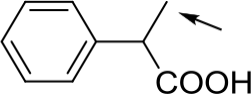	0.047^c^	23.5	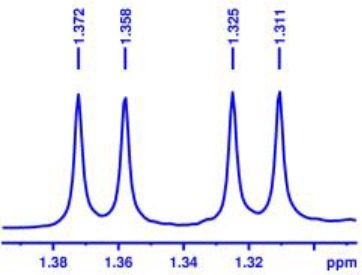
9	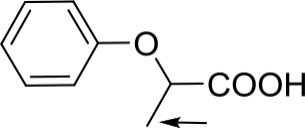	0.050^c^	25.0	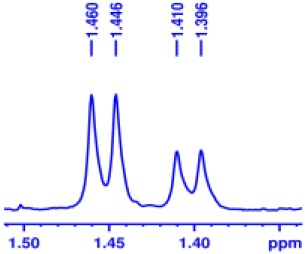
10	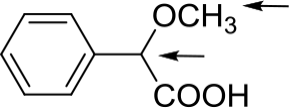	0.092	46.0	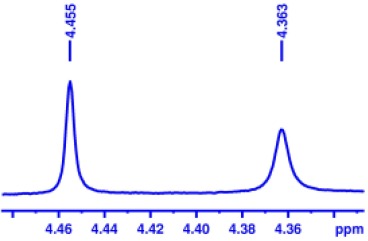
		0.041^c^	20.5	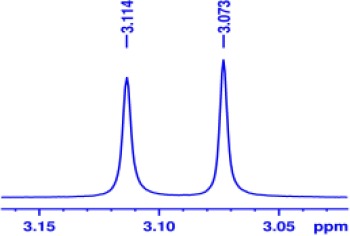
11	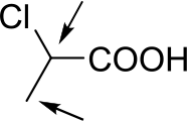	0.086	43.0	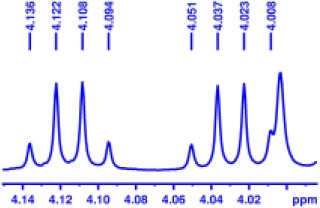
		0.033^c^	16.5	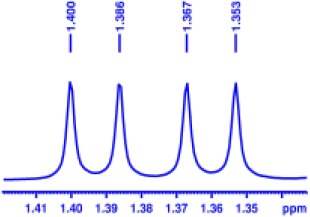
12	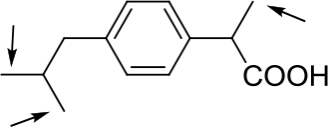	0.033^c^	16.5	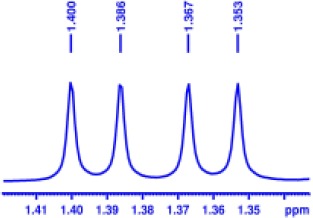
		0.013^d^	6.5	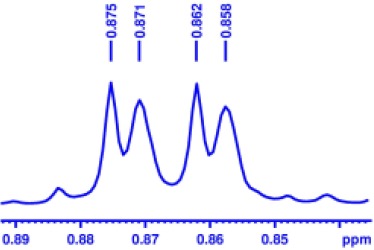
13	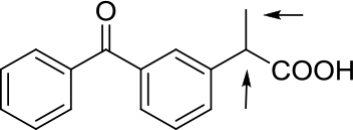	0.099	49.5	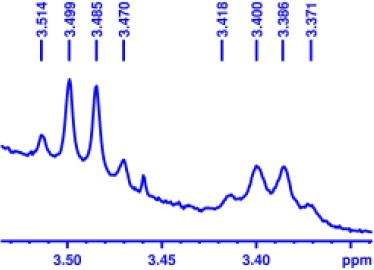
		0.052^c^	26.0	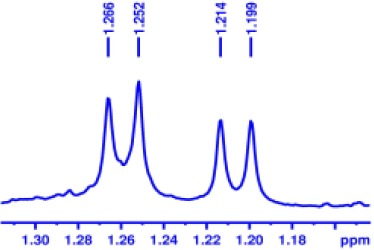
14	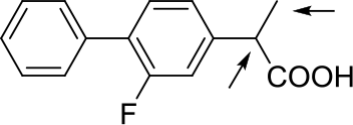	0.086	43.0	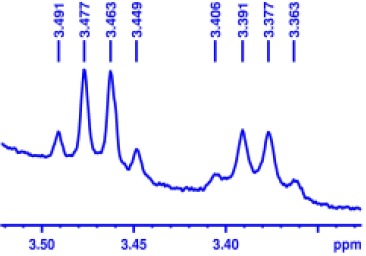
		0.067^c^	33.5	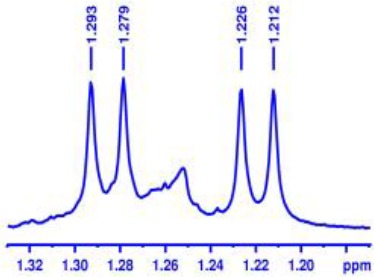

a *All analytes were prepared by mixing 2:1 of the host **3** with various carboxylic acids in NMR tubes (20 mM host and 10 mM guests in CDCl_3_)*.

b *Chemical shift non-quivalences of the methine group*.

c *Chemical shift non-equivalences of the α-methyl group*.

d *Chemical shift non-equivalences of the α-methyl protons of the isopropyl group*.

Non-steroidal anti-inflammatory drugs (NSAIDs) are the most frequently used for patients with low-back pain and inflammation. Among these phenylacetic acid analogs, the BINOL-amino alcohol **3** also exhibited clear and good chiral discrimination of signals for NSAIDs. The signals of α-CH_3_ ibuprofen, ketoprofen, and flurbiprofen were large enough with peaks identifiable (Table 1, entries 12–14 and Figures S22–S24).

**Figure 2:**
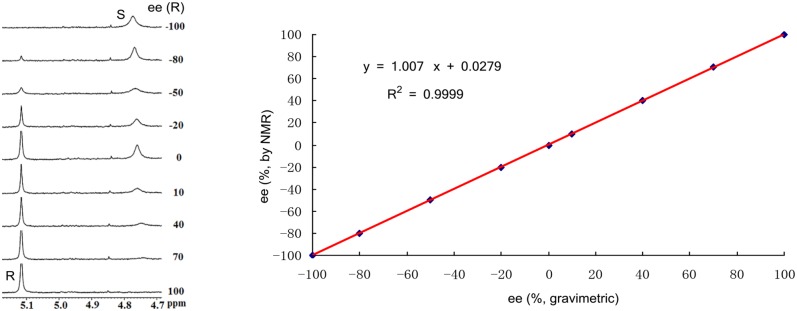
Selected overlaid partial ^1^H NMR spectra of nine different optical purities ortho-chloro-MA samples (ee% = *R*% – *S*%) in the presence of 2 equiv. BINOL-amino alcohol **3** (left); its linear correlation between the observed (*y*) and theoretical ee% values (*x*) of ortho-chloro-mandelic acid (right).

Finally, encouraged by above good enantiodiscriminating results, and to explore the practical quantitative applicability of BINOL-amino alcohol **3** for enantiomeric determination of various non-racemic samples, nine non-racemic samples containing ortho-chloro-MA with 0, 10, 40, 70, 100, −20, −50, −80, and −100% ee values were accurately calculated by integration of α-H signals of ortho-chloro-MA in ^1^H NMR analysis. The results are shown in Figure 2. The linear relationship between the NMR-determined values (*y*) and those gravimetry-determined values (*x*) is excellent with *R*^2^ = 0.999 (*y* = 1.007*x* + 0.0279, *R*^2^ = correlation coefficient).

## Conclusions

In summary, a new chiral amino alcohol containing BINOL subunit had been prepared in a five-step sequence and enantiomerically pure form starting from commercially available (R)-BINOL, The CSA **3**, which was a successfully solvating agent that was effective for carboxylic acids including some NSAIDs. In the presence of two equivalent of BINOL-amino alcohols, carboxylic acid racemates showed the chemical shift non-equivalences (ΔΔδ) large enough for the discrimination of the enantiomers (up to 320.5 Hz). Furthermore, excellent split signals were revealed in ^1^H NMR spectroscopy. The quantitative applicability of CSA **3** for enantiomeric determination of non-racemic samples was also explored based on the integration of α-H signals.

## Data Availability Statement

All datasets generated for this study are included in the article/Supplementary Material.

## Author Contributions

XL conceived the project and supervised the study. MM and GL conducted the experiments and characterized the samples. GL wrote the draft manuscript and prepared the supporting information. All authors listed have made a substantial, direct and intellectual contribution to the work, and approved it for publication.

## Conflict of Interest

The authors declare that the research was conducted in the absence of any commercial or financial relationships that could be construed as a potential conflict of interest.
