# Supplementary material for: Efficient Enantiodifferentiation of Carboxylic Acids Using BINOL-Based Amino Alcohol as a Chiral NMR Solvating Agent
Source: Front Chem. 2020 May 4;8:336. doi: 10.3389/fchem.2020.00336 (PMC7213237; doi:10.3389/fchem.2020.00336)
Supplement: Supplementary file 1 [file Data_Sheet_1.PDF]

Supporting Information

Efficient Enantiodifferentiation of Carboxylic Acids Using BINOL-based Amino Alcohol as a Chiral NMR Solvating Agent

Gaowei Li [‡1, 2], Minshan Ma [‡ 1], Guifang Wang [1], Xiaojuan Wang [2], Xinxiang Lei [1 *]

[1] School of Pharmaceutical Sciences, South Central University for Nationalities, Wuhan, 430074, China
[2] College of Chemistry and Chemical Engineering, Shangqiu Normal University, Shangqiu, 476000, China

[‡] These authors contributed equally

*** Correspondence:**
Xinxiang Lei
xxlei@mail.scuec.edu.cn (X. Lei)

Contents

**1**	**General Methods**	**S2**
**2**	**Synthesis of the chiral solvating agent 3**	**S2**
**3**	**Determination of enantiomeric purity of mandelic acid**	**S4**
**4**	**Discrimination ability of CSA 3 toward racemic guests 1-14**	**S5**
**5**	**NMR Spectra**	**S6**

1. General Methods

Solvents were dried with standard methods and freshly distilled prior to use if needed. All reactions sensitive to air or moisture were carried out under nitrogen using standard Schlenk and vacuum line techniques. CSA **3** was prepared from commercial (R)-BINOL, others chemicals were either purchased or purified by standard techniques. Melting pointswere obtained with a Yuhua X-5 micromelting point apparatus and uncorrected. Optical rotation was tested in PolAAr-3005 polarimeter and uncorrected. ^{1}H NMR and ^{13}C NMR spectra were measured on 500 MHz Brucker spectrometer in $CDCl_3$ solutions with tetramethylsilane (TMS). *J* values are given in Hz. Column chromatography was performed using Silica gel (300-400 mesh).

2. Synthesis of the chiral solvating agent 3

The chiral mono-substituted BINOL-amino alcohol **3** can be readily carried out in a five step sequence from commercially available (R)-BINOL as shown in Scheme S1. The key BINOL monoaldehyde **2** was readily generated by lithiation, acylation of the bisprotected BINOL and cleavage of the MOM ethers, the requisite monoaldehyde was condensed with ready D-plenylglycinol and followed by reduction with $NaBH_4$. The 3-monosubstituted BINOL-amino alcohol **3** was obtained as a yellow solid in 89% yield. Intermediate compound was prepared according to previously reported method. [S1, S2]

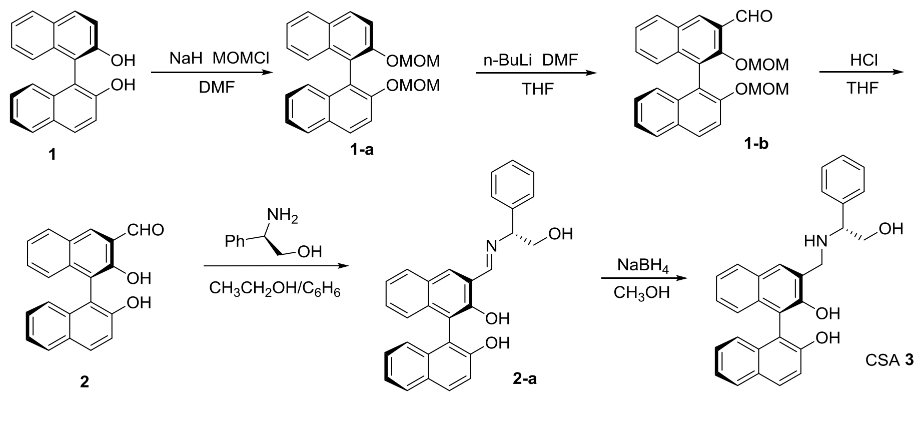

Scheme S1. Preparation and structures of 3-monosubstituted BINOL-amino alcohol **3**.

2.1. Synthetic procedures of compound 1-a

A solution of (R)-BINOL **1** (5.726 g, 20 mmol) in DMF (50 mL) was added to a stirred suspension of hydride (3.362 g, 150 mmol) at 0 ℃. After stirring for 10 min, methoxymethyl chloride (6 ml, 80 mmol) was added, and the reaction was then stirred for 50 min at 0 ℃ and 3 h at room temperature. TLC showed that the raw material response completely. The reaction mixture

was quenched with water (150 mL) and extracted with ethyl acetate (3×50 mL). The combined extracts were washed with water (3×15 mL), saturated $NaHCO_3$, brine and dried over Na_2SO_4, concentrated under reduced pressure to afford 6.74 g (90%) of crude product which can be used directly without further purification.

1-a: white solid. ^1H NMR (500 MHz, TMS, $CDCl_3$) δ 3.12 (s, 6H), 4.95 (d, J = 6.8 Hz, 2H), 5.06 (d, J = 6.8 Hz, 2H), 7.14 - 7.33 (m, 6H), 7.56 (d, J = 9.0 Hz, 2H), 7.84 (d, J = 8.2 Hz, 2H), 7.92 (d, J = 9.0 Hz, 2H); ^{13}C NMR (125 MHz, $CDCl_3$) δ 152.6, 134.0, 129.8, 129.3, 127.8, 126.2, 125.5, 124.0, 121.2, 117.2, 95.1, 55.7.

2.2. Synthetic procedures of compound 1-b

n-Butyllithium (2.5 M in hexane, 10 mL) was added to a solution of **1-a** (5.991 g, 15 mmol) in THF (50 mL) under a nitrogen atmosphere at -20 ℃. The mixture was stirred for 2 h at room temperature, which produced a grey suspension, when the reaction was cooled to 0 ℃ and DMF (2.3 mL) was added. The reaction was allowed to warm to room temperature and stirred for 10 h. The reaction mixture was quenched with water (150 mL) and extracted with ethyl acetate (3×50 mL). The combined extracts were washed with water (×3), saturated $NaHCO_3$, brine and dried over Na_2SO_4. The solvent was removed in vacuo. The crude product was recrystallized under conditions of methanol: petroleum ether (1:4). The precipitated solid was filtered, washed with petroleum ether to give a yellow solid product 3.079 g (57%).

1-b: yellow solid. ^1H NMR (500 MHz, $CDCl_3$) δ 2.87 (s, 6H), 4.69 (d, J 6.6 Hz, 2H), 4.73 (d, J =6.3 Hz, 2H), 7.22 (d, J= 8.7 Hz, 2H), 7.42-7.50 (m, 2H), 7.52-7.60 (m, 2H), 8.08 (d, J =8.1 Hz, 2H), 8.62 (s, 2H), 10.55 (s, 2H); ^{13}C NMR (125 MHz, $CDCl_3$) δ 57.0, 100.6, 125.9, 126.1, 126.3, 128.8, 129.6, 130.1, 130.3, 132.29, 136.7, 154.0, 190.6.

2.3. Synthetic procedures of compound 2

Compound **1-b** (2.012 g, 5 mmol) was dissolved in THF (30 mL). The solution was cooled in an ice bath, and then 12 M hydrochloric acid (0.5 mL) was added over 5 min. The ice bath was removed and the reaction mixture was stirred for 3 h at room temperature. TLC showed the starting material the reaction was complete. The reaction mixture was quenched with water (150 mL). The solution was extracted with ethyl acetate (3 × 50 mL), the combined extracts were washed with water, saturated $NaHCO_3$, brine and dried over Na_2SO_4. The solvent was removed in vacuo and the residue purified by column chromatography on silica gel eluting with petroleum ether /ethyl acetate

(20:1) to give soft yellow solid 1.410 g (90%).

2: soft yellow solid. ^{1}H NMR (500 MHz, $CDCl_3$) δ 5.06 (s, 1H), 7.21 - 7.43 (m, 6H), 7.07 (d, J = 8.4 Hz, 1H), 7.86 (d, J = 8.0 Hz, 1H), 7.91 (d, J = 8.9 Hz, 1H), 7.95 - 7.99 (m, 1H), 8.32 (s, 1H), 10.12 (s, 1H), 10.61 (s, 1H); ^{13}C NMR (125 MHz, $CDCl_3$) δ 196.6, 154.4, 151.5, 139.1, 137.7, 133.4, 131.2, 130.5, 130.1, 129.3, 128.3, 127.8, 126.7, 125.0, 124.4, 123.5, 122.1, 117.7, 115.1, 113.2.

2.4. Synthetic procedures of compound 2-a

A solution of D-Phenylglycinol (0.274 g, 2 mmol) in ethanol (5 mL) and benzene (5 mL) was added to compound **2** (0.628 g, 2 mmol). The reaction mixture was heated in a Dean-Stark apparatus for 10 h. TLC showed the starting material the reaction was complete. Concentrated to give a yellow-orange solid (0.850 g, yield 98%).

2-a: yellow-orange solid. m.p.113-114 ^{o}C; $[\alpha]^{25}_{D}$ = +202.5 (c 0.24, CH_2Cl_2); ^{1}H NMR (500 MHz, TMS, $CDCl_3$) δ 3.87 (d, J = 6.5 Hz, 2H), 4.53 (t, J = 6.5 Hz, 1H), 5.15 (s, 1H), 7.13 - 7.18 (m, 2H), 7.23 - 7.39 (m, 10H), 7.96 - 7.84 (m, 3H), 8.03 (s, 1H), 8.74 (s, 1H), 13.30 (s, 1H); ^{13}C NMR (125 MHz, $CDCl_3$) δ 166.1, 155.4, 151.5, 138.8, 135.5, 135.1, 133.5, 130.2, 129.3, 129.0, 128.3, 128.1, 127.7, 127.2, 126.6, 124.7, 124.13, 123.4, 120.8, 117.7, 114.3, 113.8, 76.2, 67.4. IR (KBr) 3504, 2358, 1629, 1503 cm^{-1}.

2.5. Synthetic procedures of CSA 3

Compound **2-a** (0.628 g, 2 mmol) was dissolved in in methanol (20 mL). The solution was cooled in an ice bath, and then excess of $NaBH_4$ (0.23 g, 6 mmol) was added, stirring for 3h. The reaction mixture was quenched with 12 M hydrochloric acid (20 mL). The solution was extracted with $CHCl_3$ (3 × 50 mL), the combined extracts were dried over Na_2SO_4. Concentrated to give a soft yellow solid 0.698 g (89%).

CSA **3**: soft yellow solid. $[\alpha]^{25}_{D}$ = -24.5 (c 0.294, CH_2Cl_2); ^{1}H NMR (500 MHz, $CDCl_3$) δ 3.87 (d, J = 6.5 Hz, 2H), 4.54 (t, J = 6.5 Hz, 1H), 5.16 (s, 1H), 7.14 - 7.19 (m, 2H), 7.24 - 7.40 (m, 10H), 7.87 - 7.94 (m, 3H), 8.04 (s, 1H), 8.75 (s, 1H), 13.30 (s, 1H); ^{13}C NMR (125 MHz, $CDCl_3$) δ 166.1, 155.4, 151.5, 138.8, 135.5, 135.1, 133.5, 130.2, 129.3, 129.0, 128.3, 128.1, 127.7, 127.2, 126.6, 124.7, 124.13, 123.4, 120.8, 117.7, 114.3, 113.8, 76.2, 67.4.

3. Determination of enantiomeric purity of mandelic acid

To determine the enantiomeric purity of the carboxylic acids, ten *o*-Cl-mandelic acid samples

with -80%, -60%, -40%, -20%, 0%, 20%, 40%, 60%, 80%, 100% *ee* were prepared at a concentration of 10 mM in $CDCl_3$, respectively, expressed as % *R* in the data. The CAS **3** was also dissolved in $CDCl_3$ at a concentration of 10 mM. Then 250 μL of CAS **3** and 250 μL of *o*-Cl-mandelic acid with different *ee*'s were mixed in the NMR tube generating a total concentration of 10 mM with a molar ratio of 1:1. Then the enantiomeric purity of the carboxylic acids was determined by ^1H NMR method. The plotting of gravimetry *ee* value (y axis) versus NMR observed *ee* value (x axis) presented excellent linearity with $R^2 = 0.9995$.

4. Discrimination ability of CSA 3 toward racemic guests 1-12

At first, CSA **3**, and the guests were separately dissolved in $CDCl_3$ with a concentration of 10 mM. Then, 0.25 mL of CSA **3** and 0.25 mL guest were added to NMR tubes, so that the total volume was 0.5 mL, and the concentration of CSA **3** and guest was 10 mM. The ^1H NMR spectra of all samples were recorded on a 500 MHz spectrometer.

5. NMR Spectra

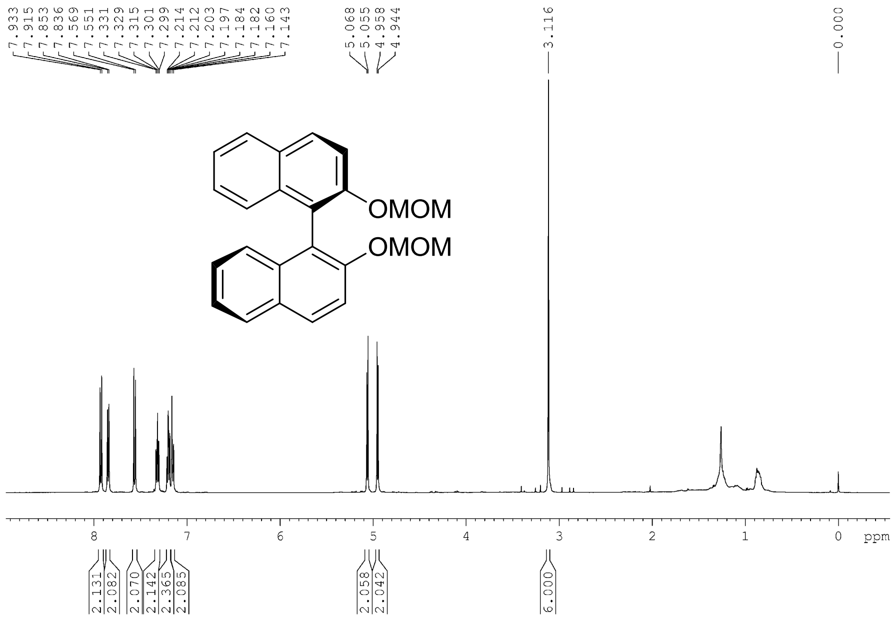

Figure S1. ^{1}H NMR (500 MHz, $CDCl_3$, TMS) of compound 1-a.

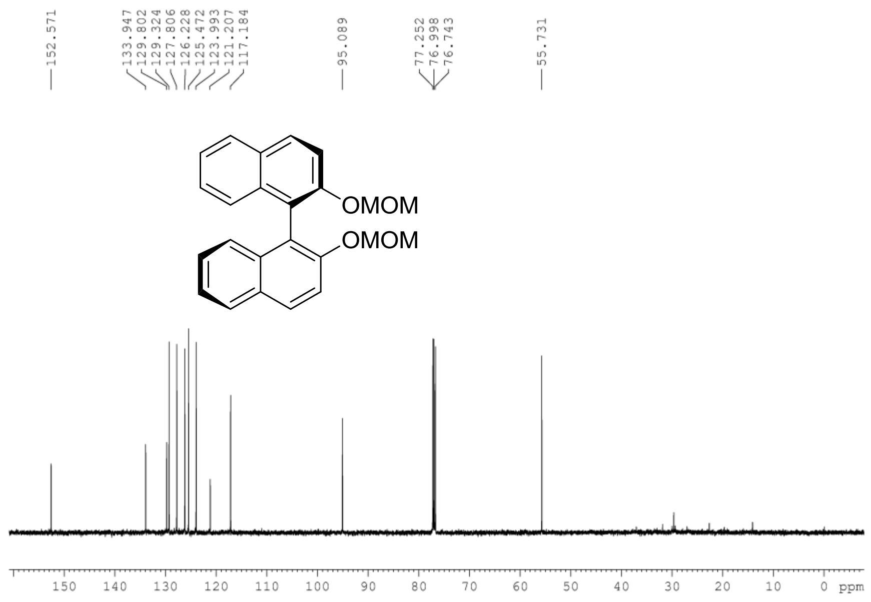

Figure S2. ^{13}C NMR (125 MHz, $CDCl_3$) of compound 1-a

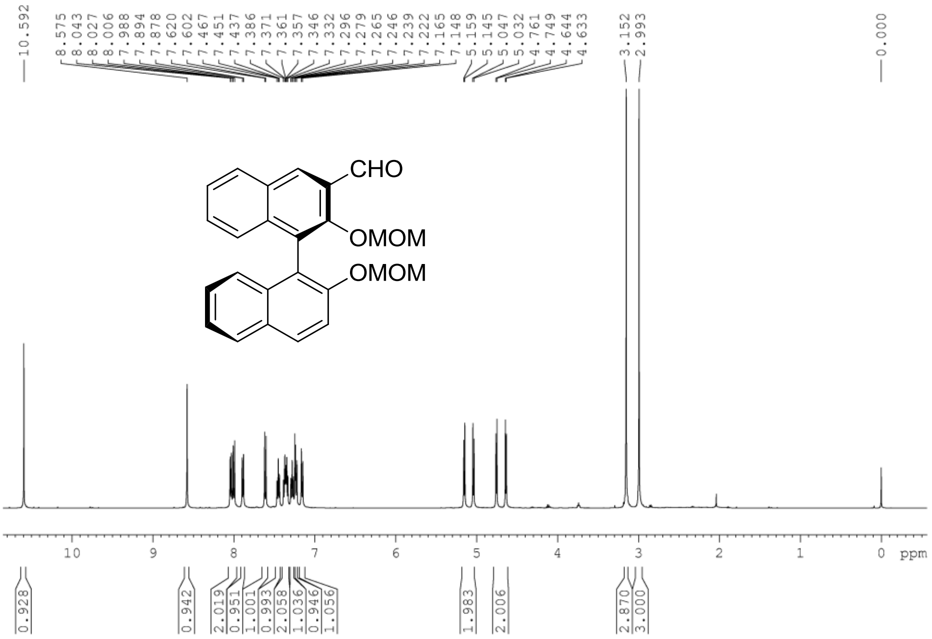

Figure S3. ^{1}H NMR (500 MHz, $CDCl_3$, TMS) of compound 1-b.

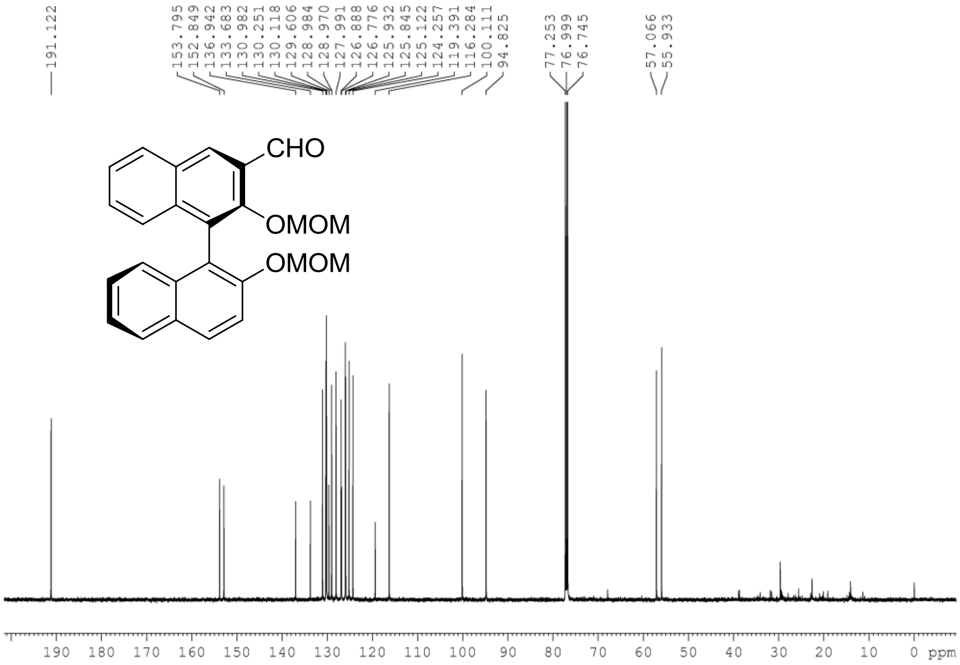

Figure S4. ^{13}C NMR (125 MHz, $CDCl_3$) of compound 1-b.

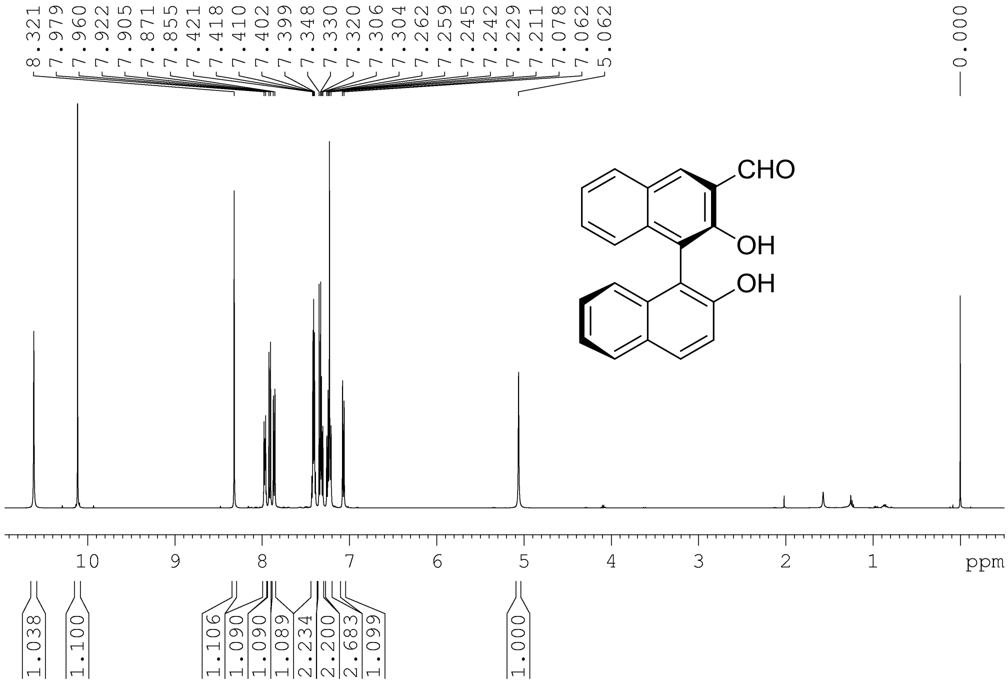

Figure S5. ^{1}H NMR (500 MHz, $CDCl_3$, TMS) of compound 2.

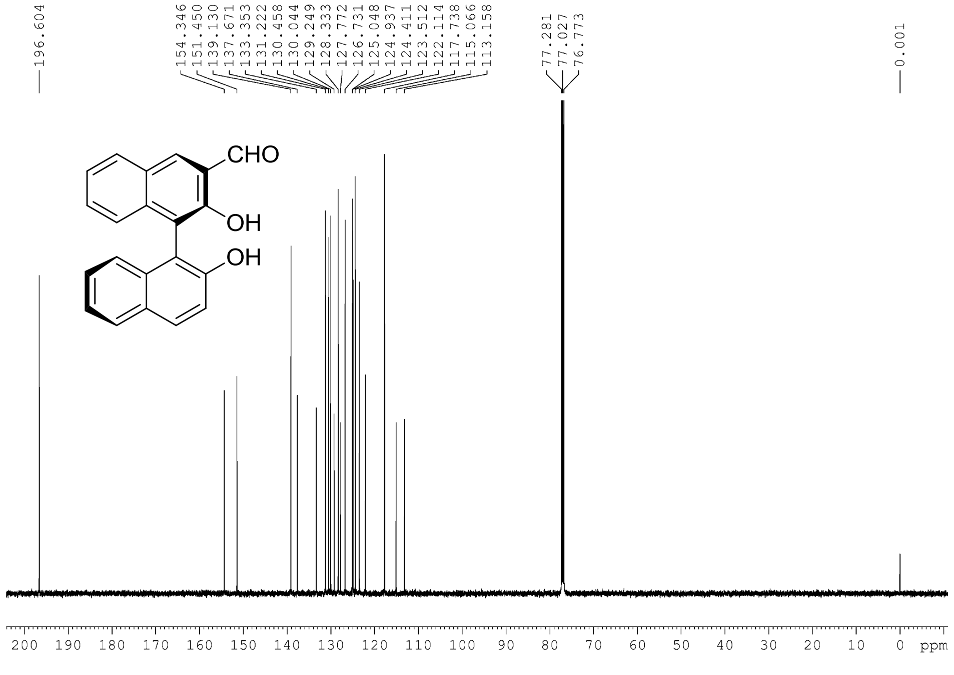

Figure S6. ^{13}C NMR (125 MHz, CDCl3) of compound 2.

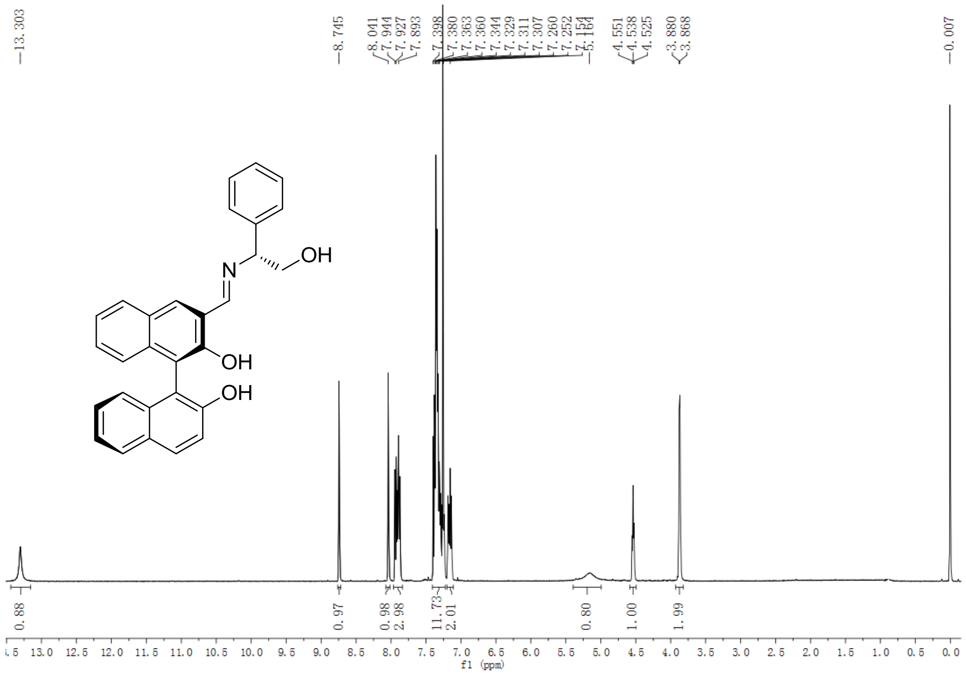

Figure S7. ^{1}H NMR (500 MHz, $CDCl_3$, TMS) of compound 2-a.

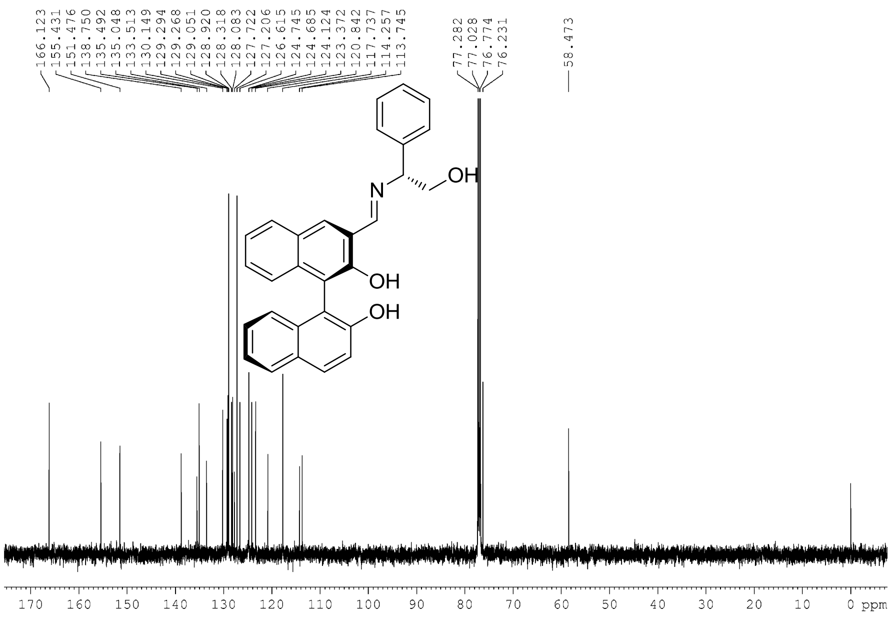

Figure S8. ^{13}C NMR (125 MHz, $CDCl_3$) of compound 2-b.

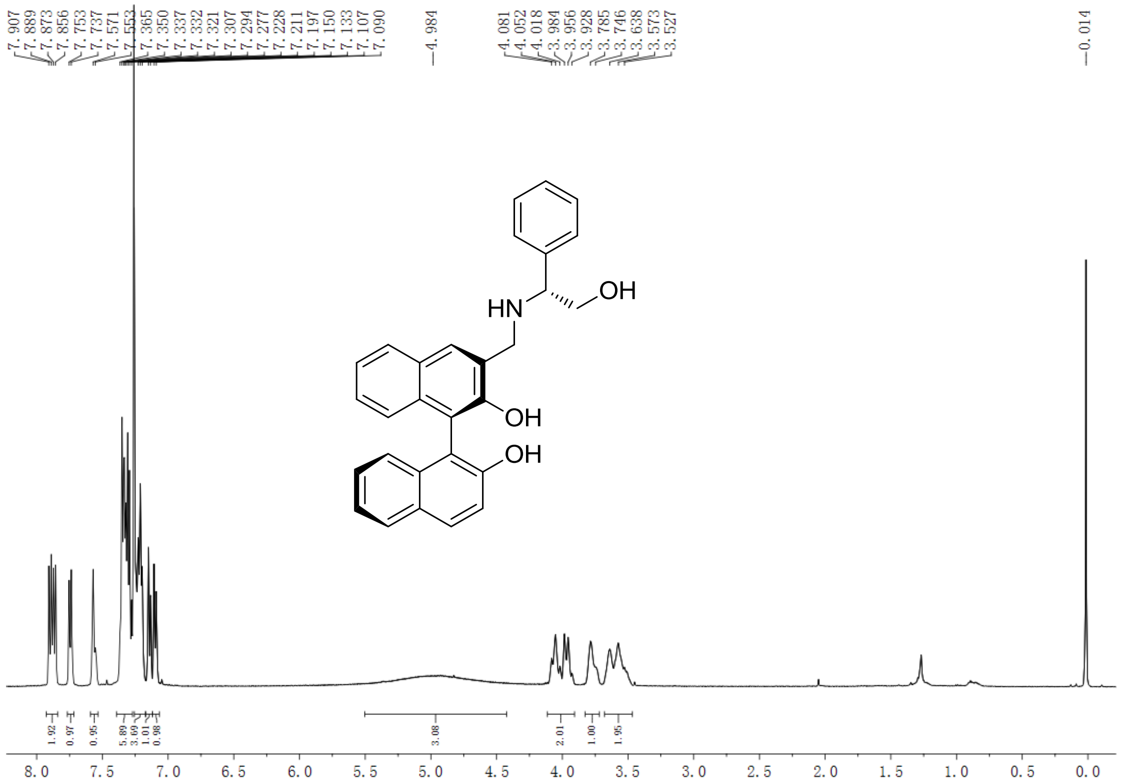

Figure S9. **^{1}H NMR (500 MHz, $CDCl_3$, TMS) of CSA 3**

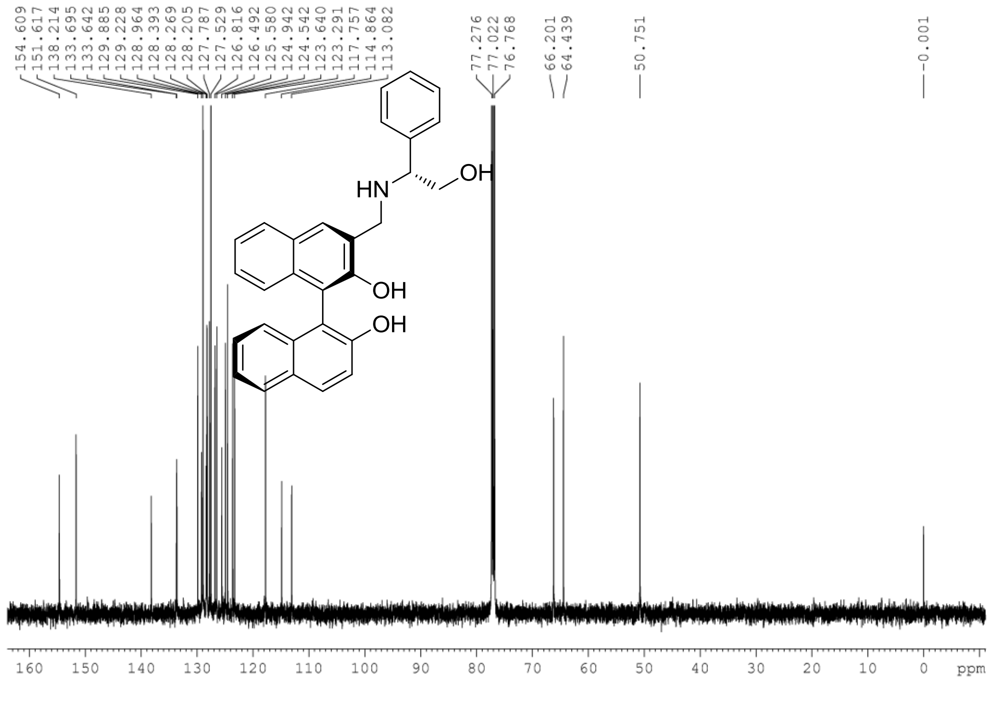

Figure S10. **^{13}C NMR (125 MHz, $CDCl_3$) of CSA 3.**

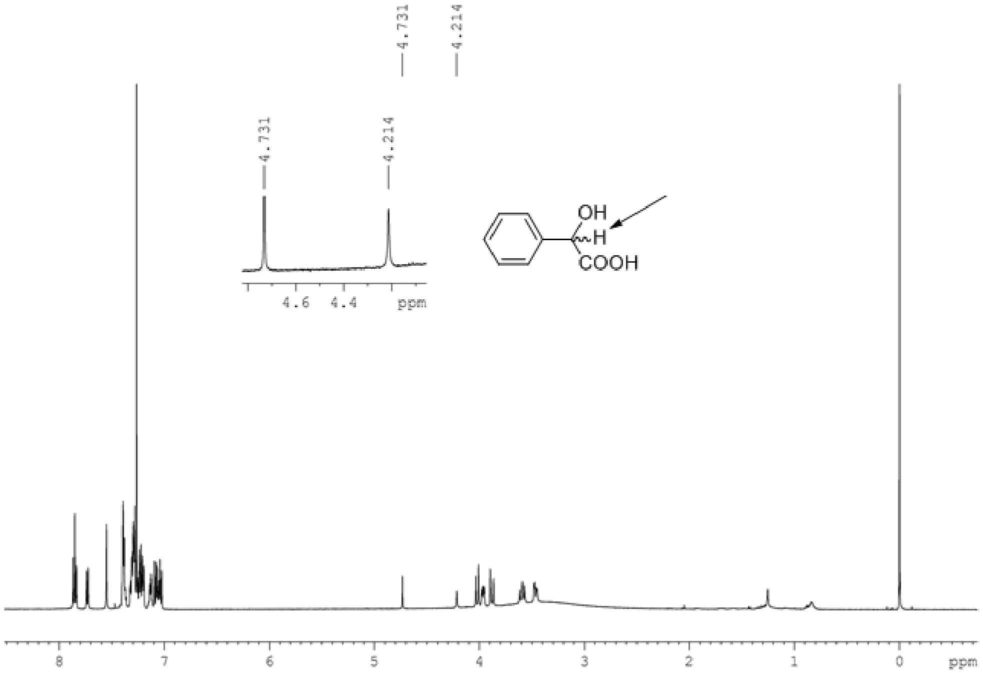

Figure S11. ^{1}H NMR Spectra of CSA and carboxylic acid 1

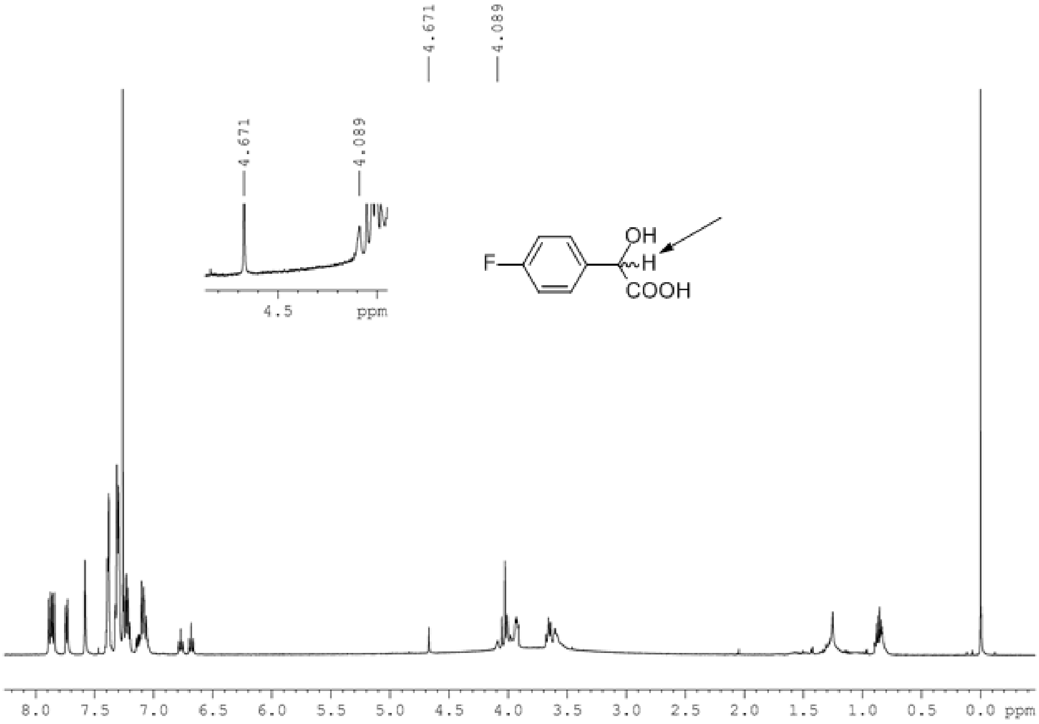

Figure S12. ^{1}H NMR Spectra of CSA and carboxylic acid 2

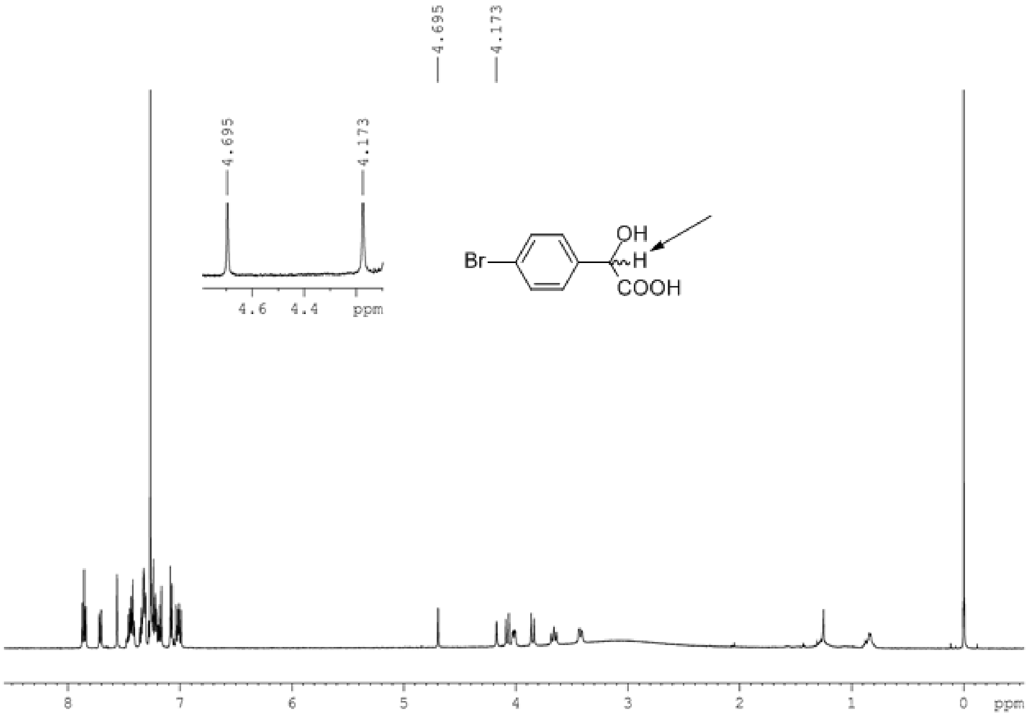

Figure S13. ^{1}H NMR Spectra of CSA and carboxylic acid 3

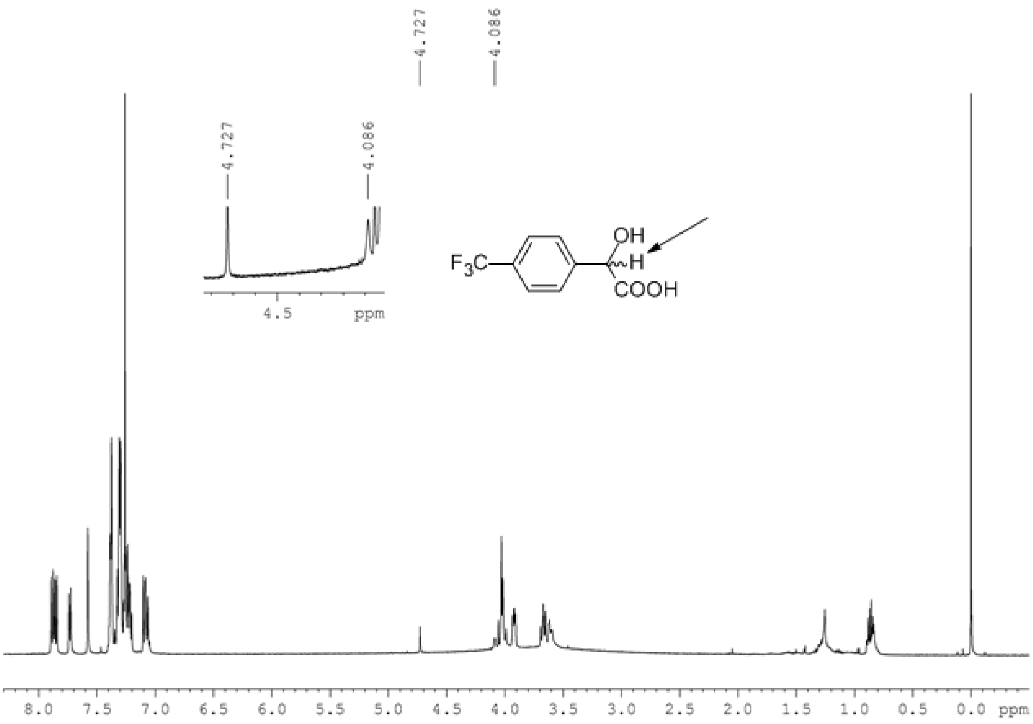

Figure S14. ^{1}H NMR Spectra of CSA and carboxylic acid 4

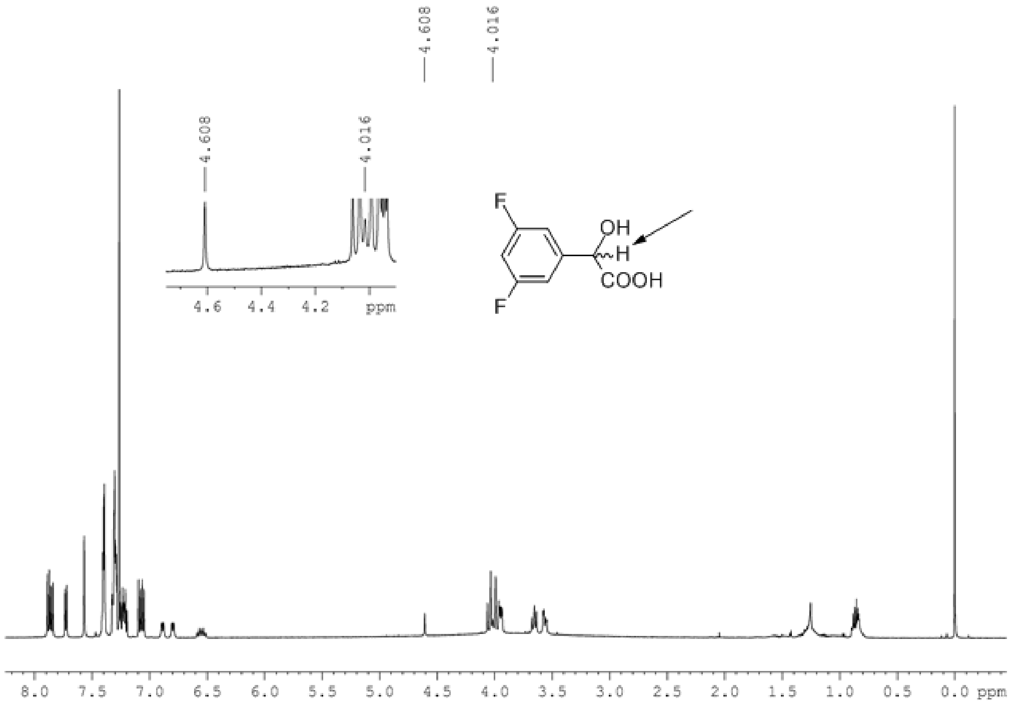

Figure S15. ^{1}H NMR Spectra of CSA and carboxylic acid 5

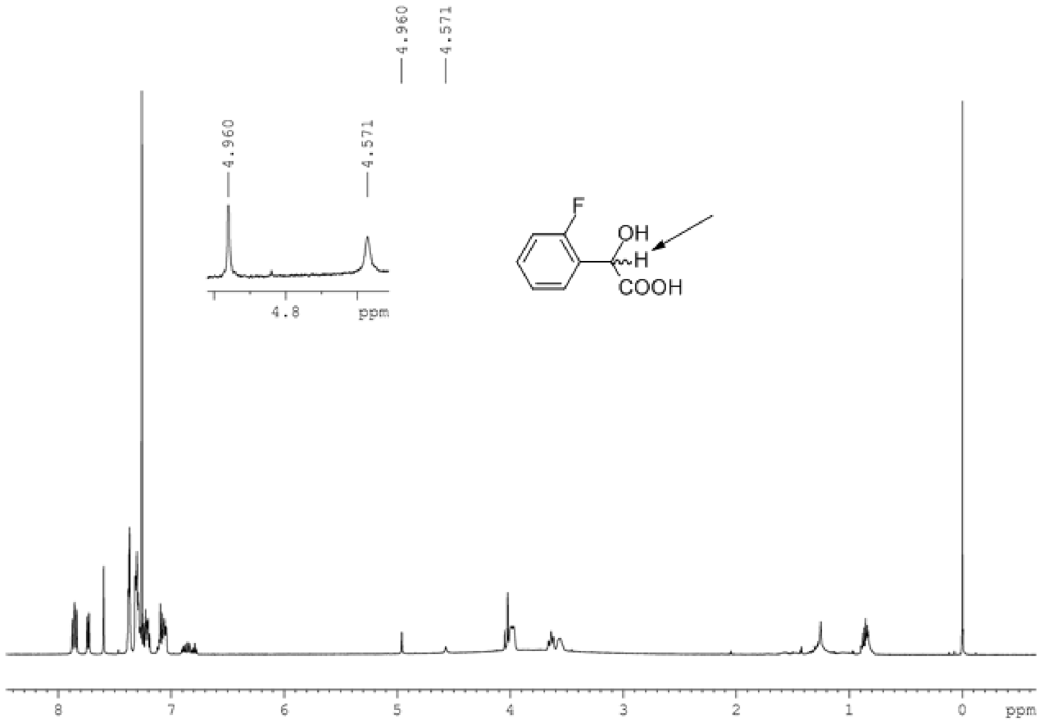

Figure S16. ^{1}H NMR Spectra of CSA and carboxylic acid 6

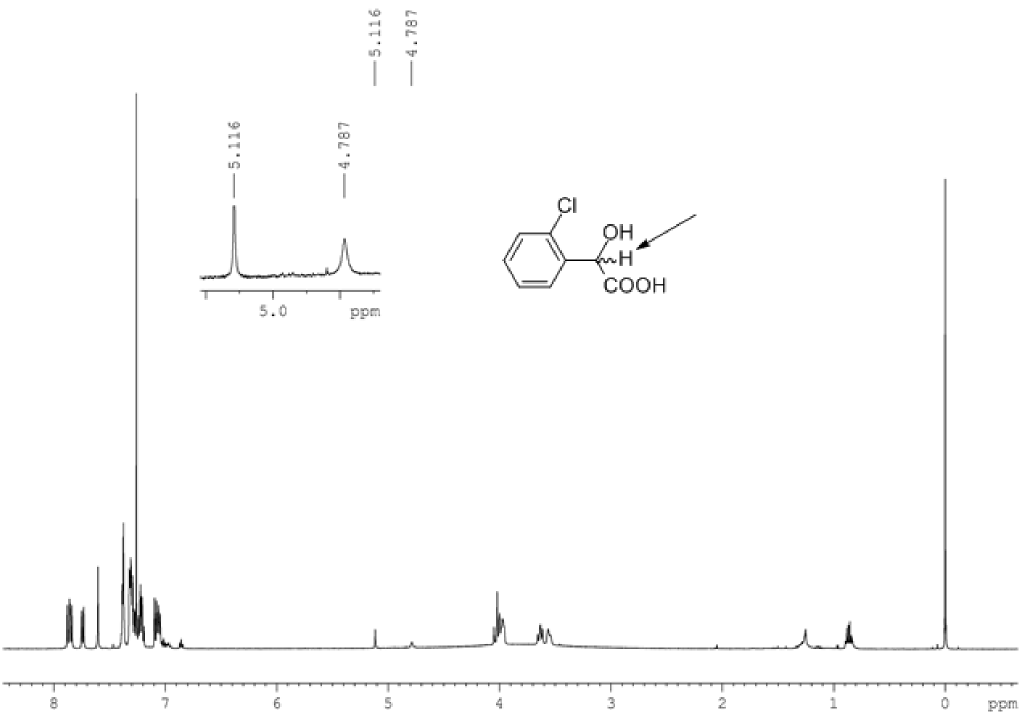

Figure S17. ^{1}H NMR Spectra of CSA and carboxylic acid 7

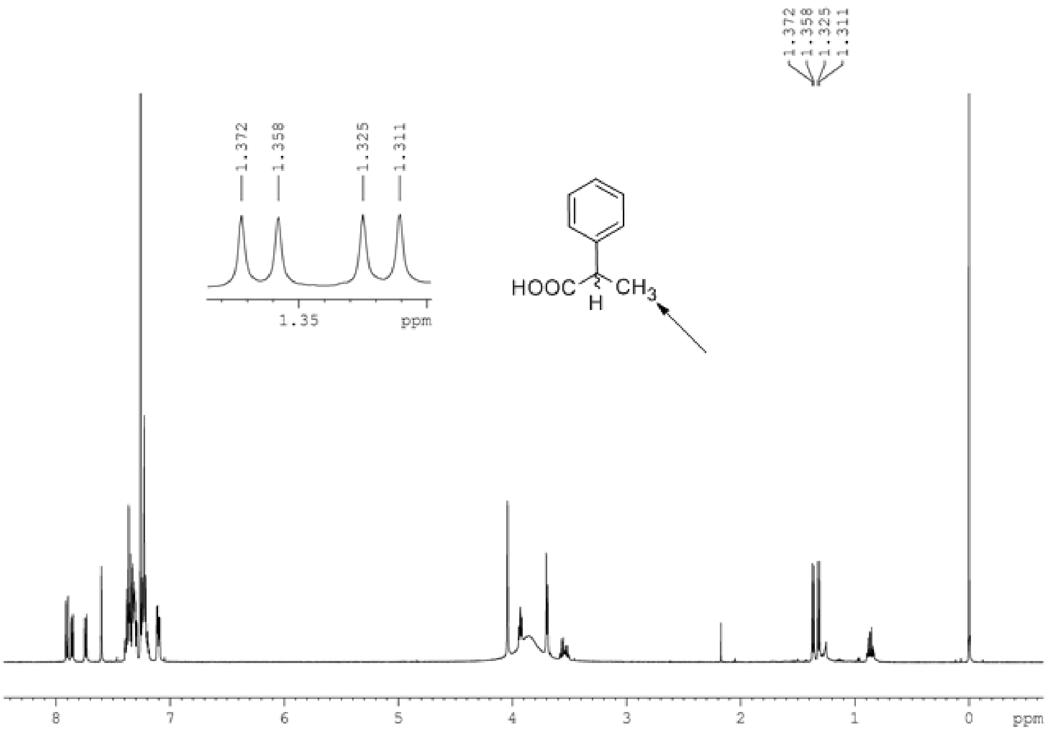

Figure S18. ^{1}H NMR Spectra of CSA and carboxylic acid 8

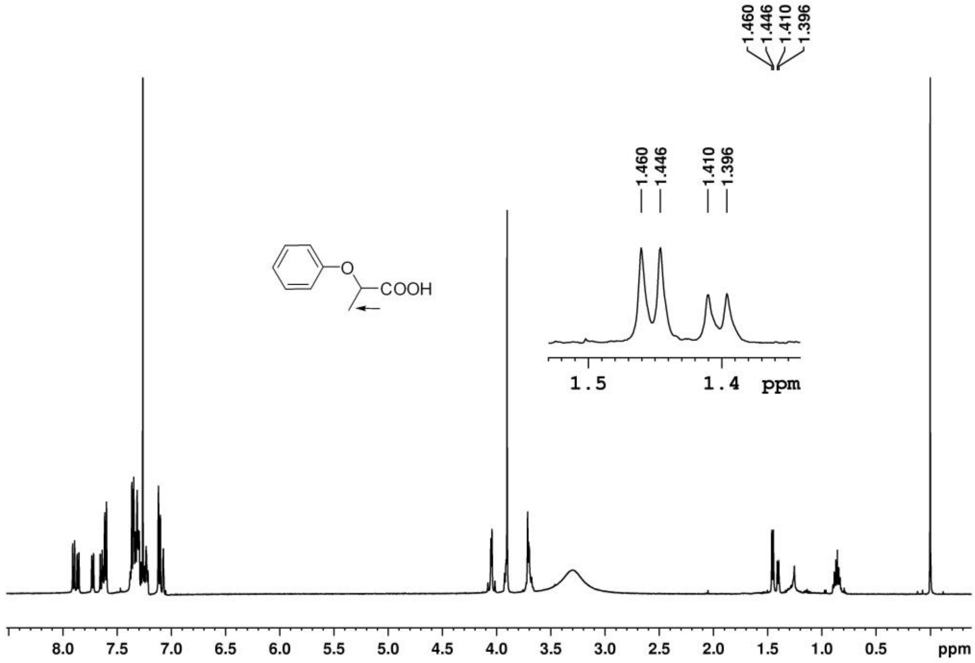

Figure S19. ^{1}**H NMR Spectra of CSA and carboxylic acid 9**

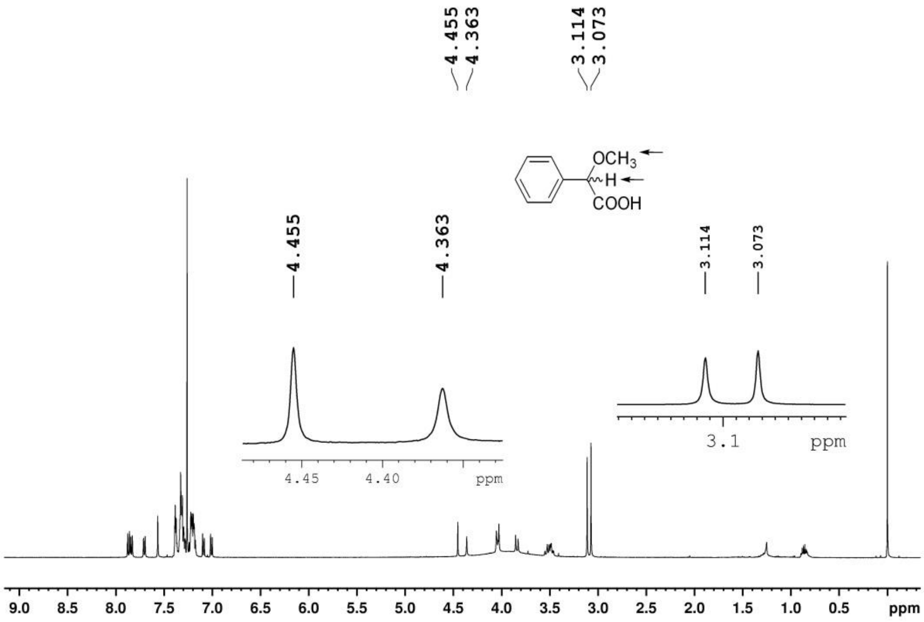

Figure S20. ^{1}**H NMR Spectra of CSA and carboxylic acid 10**

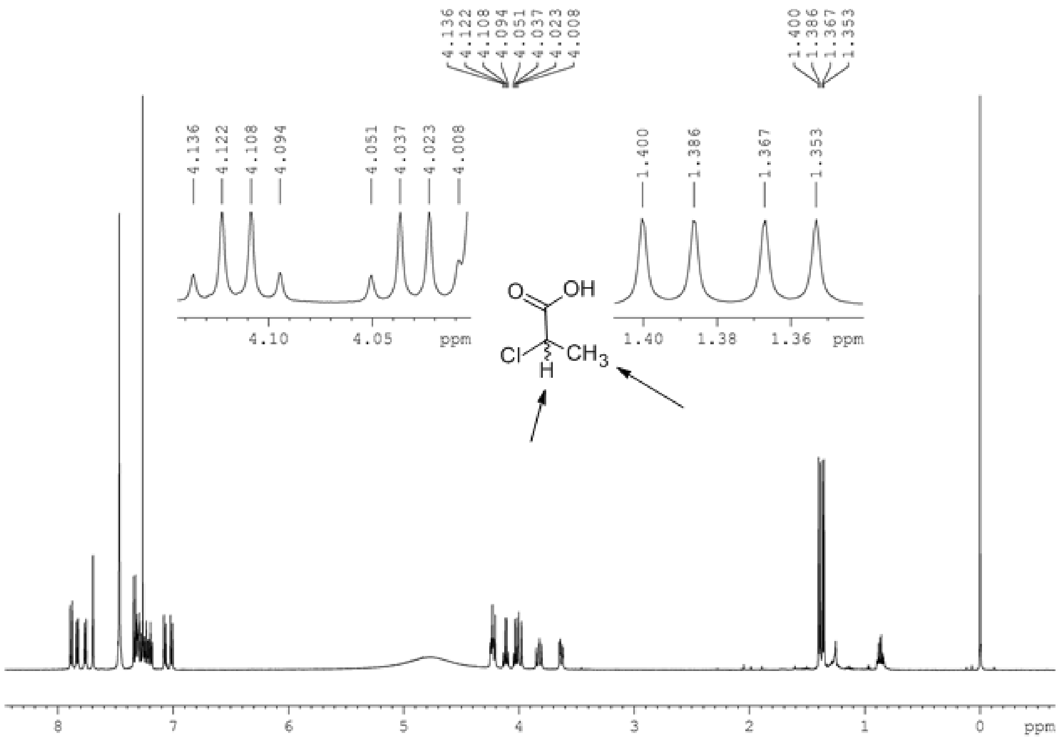

Figure S21. ^{1}H NMR Spectra of CSA and carboxylic acid 11

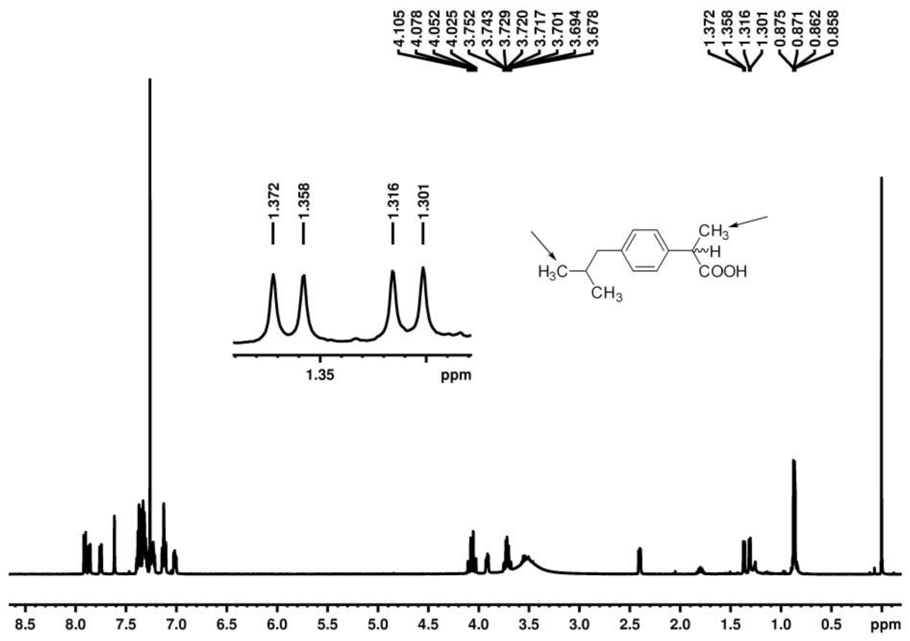

Figure S22. ^{1}H NMR Spectra of CSA and carboxylic acid 12

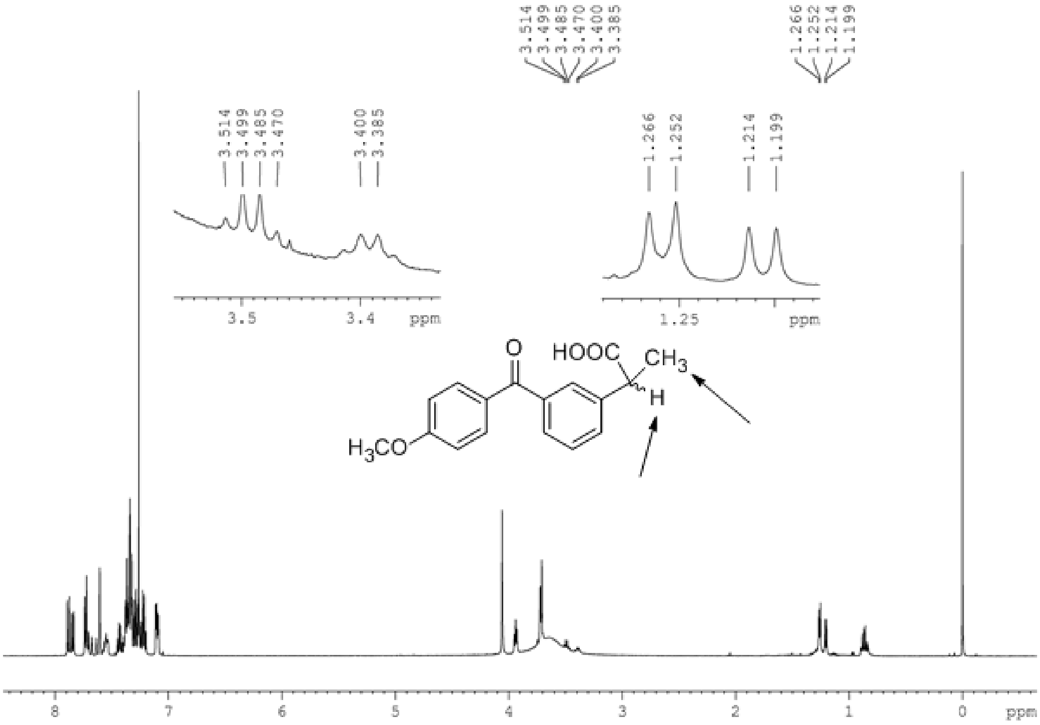

Figure S23. ^{1}H NMR Spectra of CSA and carboxylic acid 13

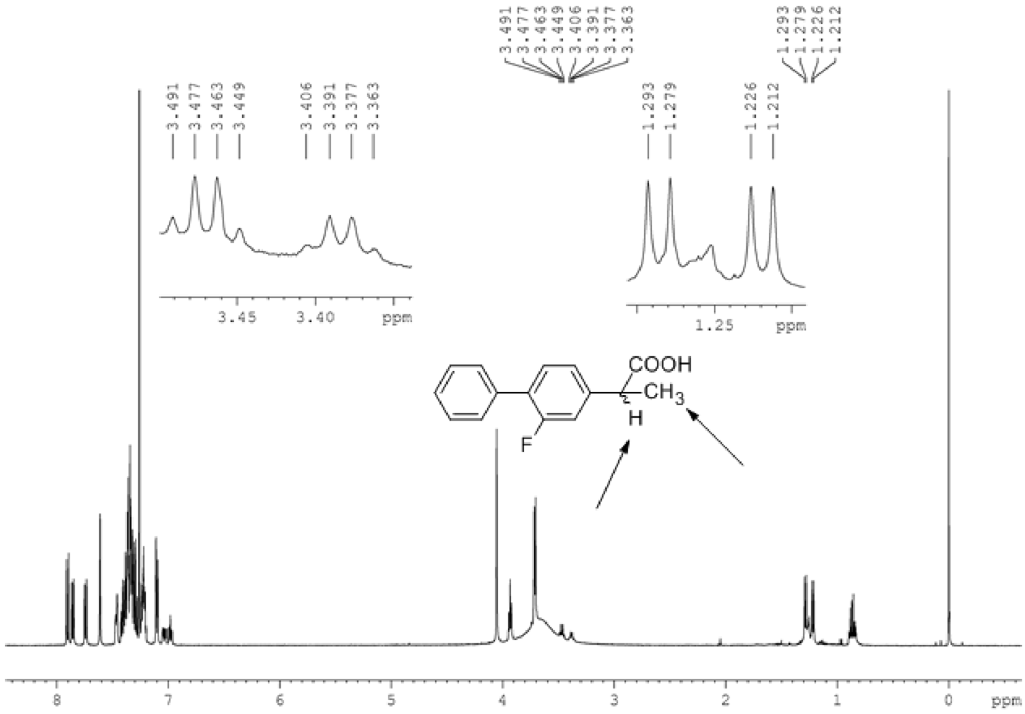

Figure S24. ^{1}H NMR Spectra of CSA and carboxylic acid 14

6. References

S1. Bougauchi, M., Watanabe, S., Arai, T., Sasai, H., and Shibasaki, M. (1997). Catalytic asymmetric epoxidation of α,β-unsaturated ketones promoted by lanthanoid complexes. *J. Am. Chem. Soc.* 119, 2329-2330. doi:10.1021/ja964037o

S2. Dong, M., Dong, Y. M., Ma, T. H., Wang, Y. W., and Peng, Y. (2012) A highly selective fluorescence-enhanced chemosensor for Al^{3+} in aqueous solution based on a hybrid ligand from BINOL scaffold and β-amino alcohol. *Inorg. Chim. Acta* 381, 137-142. doi:10.1016/j.ica.2011.08.043
